# Low Prefrontal GABA Levels Are Associated With Poor Cognitive Functions in Professional Boxers

**DOI:** 10.3389/fnhum.2019.00193

**Published:** 2019-06-11

**Authors:** Geon Ha Kim, Ilhyang Kang, Hyeonseok Jeong, Shinwon Park, Haejin Hong, Jinsol Kim, Jung Yoon Kim, Richard A. E. Edden, In Kyoon Lyoo, Sujung Yoon

**Affiliations:** ^1^Ewha Brain Institute, Ewha Womans University, Seoul, South Korea; ^2^Department of Neurology, Ewha Womans University College of Medicine, Seoul, South Korea; ^3^Department of Brain and Cognitive Sciences, Ewha Womans University, Seoul, South Korea; ^4^Department of Radiology, Incheon St. Mary’s Hospital, College of Medicine, The Catholic University of Korea, Seoul, South Korea; ^5^Division of Neuroradiology, Russell H. Morgan Department of Radiology and Radiological Science, The Johns Hopkins University School of Medicine, Baltimore, MD, United States; ^6^F.M. Kirby Center for Functional Brain Imaging, Kennedy Krieger Institute, Baltimore, MD, United States; ^7^College of Pharmacy, Graduate School of Pharmaceutical Sciences, Ewha Womans University, Seoul, South Korea

**Keywords:** GABA, professional boxers, prefrontal cortex, cognitive impairment, traumatic brain injury

## Abstract

Cognitive dysfunction has long been recognized as a frequently observed symptom in individuals with repetitive mild traumatic brain injury (rmTBI) such as professional boxers. The exact neurobiological mechanisms underlying this cognitive deficit have not yet been identified, but it is agreed upon that the prefrontal cortex (PFC) is one of the most commonly affected brain regions in professional boxers. Noting the pivotal role of the two major brain metabolites in human cognitive functions, γ-aminobutyric acid (GABA) and glutamate/glutamine (Glx), we hypothesized that alterations in levels of GABA and Glx in the PFC would be prominent and may correlate with cognitive deficits in professional boxers. Twenty male professional boxers (Boxers) and 14 age-matched healthy males who had never experienced any TBI (CON) were recruited. Using a 3T magnetic resonance imaging (MRI) scanner, single-voxel proton magnetic resonance spectroscopy with Mescher-Garwood point-resolved spectroscopy (MEGA-PRESS) sequence was performed to evaluate the levels of GABA and Glx in the PFC. Cognitive function was assessed using the memory and attention domains from the Cambridge Neuropsychological Test Automated Battery. The Boxers showed lower GABA level in the PFC compared to the CON, while also showing lower performance in the attention and memory domains. There were no significant between-group differences in Glx levels. Furthermore, the GABA level correlated with memory performance in the Boxers, but not in attention performance. The current findings may suggest that alterations in GABA levels in the PFC may be a potential neurochemical correlate underlying memory dysfunction related to rmTBI.

## Introduction

The incidence of sports-related traumatic brain injury (TBI) continues to increase. Research indicates that approximately 1.6–3.8 million individuals overall suffer from TBI each year in the USA (Langlois et al., 2006; Nagahiro and Mizobuchi, 2014), where the incidence rate is particularly high for those who play contact sports. In fact, this could be an underestimation considering that approximately 50% of all sports-related concussions have not been reported (McCrea et al., 2004; Williamson and Goodman, 2006; Harmon et al., 2013). As many athletes fell victim to this “silent epidemic” that stems from the reluctance to report TBI within athletic world, it has been receiving greater attention in the media as well as the medical and scientific fields (Kulbe and Geddes, 2016). Specifically, chronic repetitive mild TBI (rmTBI) in relation to contact sports has been of particular interest, as it may be associated with an increased risk of developing dementia in later life (Martland, 1928; Guskiewicz et al., 2005).

Professional boxing is one of the most commonly studied contact sports in the field of rmTBI (Bernick and Banks, 2013). Close associations between professional boxing and the occurrence of chronic traumatic encephalopathy (CTE), a type of dementia in later life, have been reported (Martland, 1928; McCrory et al., 2007; McKee et al., 2013). The development of dementia in later life among professional boxers may result from the combined damage of cumulative boxing-related rmTBI as well as age-associated neurodegenerative injuries on the brain (Heilbronner et al., 2009; Smith D. H. et al., 2013). Therefore, in order to develop the early preventive strategies for rmTBI, it would be important to investigate the subtle and silent brain alterations related to rmTBI, by recruiting currently active young professional boxers who are clinically asymptomatic. In addition, early detection of brain changes related to rmTBI among active professional boxers could be helpful in providing appropriate preventive intervention for professional boxers who are at a higher risk of dementia (Wilde et al., 2016). Considering that mTBI-related deficits in attention and memory domains have been frequently observed in professional boxers (Roberts, 1969; Jordan et al., 1996) as well as in young-to-middle-aged amateur athletes with rmTBI (List et al., 2015), investigation of alterations in these cognitive domains enable us to identify cognitive dysfunctions before clinical manifestations are evident especially among young-to middle-aged professional boxers.

A few previous translational studies have suggested that neurotransmitters including acetylcholine, glutamate, dopamine, serotonin, and γ-aminobutyric acid (GABA) may be potential biomarkers of cognitive dysfunctions in TBI (Sun and Feng, 2014). However, there are currently no clinically available biomarkers to identify cognitive deficits that are due to neuronal dysfunction as a result of rmTBI. Of the neurotransmitters mentioned above, the GABA, as a primary inhibitory neurotransmitter in the central nervous system (CNS), have shown to play a role in attention and memory function (Sun and Feng, 2014; Guerriero et al., 2015). Since recent technical advancements in proton magnetic resonance spectroscopy (^1^H-MRS) have permitted further investigation with regards to real-time cerebral metabolism including GABA (Edden et al., 2014; Mullins et al., 2014) in the human brain, several previous studies have shown that lower GABA levels were also associated with cognitive deficits as well as increased risks of dementia in older adults (Bañuelos et al., 2014; Porges et al., 2017).

However, only a few studies have been performed to investigate the alterations in the GABA levels of the human brain in relation to mTBI (Tremblay et al., 2014; Wilke et al., 2017) and did not detect significant group-differences in GABA concentration between the patients with chronic mTBI and the healthy control subjects. The lack of alterations in GABA concentration in patients with mTBI from the previous studies could be derived from the location of voxel of interest (VOI), the primary motor cortex (Tremblay et al., 2014; Wilke et al., 2017). As memory and attention dysfunctions typically precede motor dysfunctions in patients with rmTBI (Stern et al., 2013), the assessment of GABA levels within the brain areas that are closely associated with these cognitive functions such as the prefrontal cortex (PFC), may yield more reliable results regarding the underlying mechanisms of rmTBI-related brain alterations in professional boxers. Furthermore, the PFC is one of the most commonly involved brain regions in professional fighters with cognitive impairment (Cazalis et al., 2011; Zhou et al., 2012; Mishra et al., 2017). Therefore, unlike previous studies applying the primary motor cortex as a VOI (Tremblay et al., 2014; Wilke et al., 2017), the current study measured GABA level in the PFC as a VOI. In addition, as GABA concentrations in the PFC has been reported to play a role in cognitive modulation (Yoon et al., 2016; Porges et al., 2017), we hypothesized that the professional boxers, relative to healthy controls, may show lower levels of GABA in the PFC and this alteration may be associated with deficits in memory or attention functions.

## Materials and Methods

### Participants

Twenty male professional boxers (hereafter referred to as Boxers) aged between 20 and 40 years and 14 age- and sex- matched healthy controls (hereafter referred to as CON) were recruited through community advertisement. All participants in the Boxers group have had at least 2 years of experience in professional boxing and had experienced mTBI and multiple subconcussive injuries. In contrast, all healthy individuals had not experienced any sort of TBI. The mTBI was defined as a brain trauma that may produce transient mental status changes which resolve spontaneously and completely in a few minutes (Carroll et al., 2004; Group, 2006; Pérez-Arredondo et al., 2016; Neidecker et al., 2019). We excluded boxers who had ever suffered from moderate to severe brain injury which is defined as resulting in a loss of consciousness for more than 20 min. The following additional exclusion criteria were used for all participants regardless of group status: (1) suspected or diagnosed major neurological or psychiatric illnesses; (2) any contraindications to magnetic resonance imaging (MRI); (3) severe visual or hearing impairments that interfere with their ability to respond to questionnaires; (4) a history of medications that could affect cognitive and emotional functions in the last 3 months prior to participation; or (5) any other major medical problems that may require immediate attention. All participants were also asked to refrain from consuming any alcohol or caffeine for 12 h prior to the MRI scanning.

All participants provided written informed consent to participate in the study. The study protocol was approved by the Institutional Review Board of Ewha Womans University.

### Clinical and Neuropsychological Assessment

Clinical assessment of participants included the evaluation of medical history as well as physical and neurological examinations. The total amount of physical activity for each participant was also measured using the International Physical Activity Questionnaire (IPAQ; Chun, 2012). The variable for physical activity was described as metabolic equivalent of task (MET)-minutes/week, which was calculated by multiplying the MET-minutes scores with the number of performance days per week. Demographic and clinical information are presented in Table 1.

**Table 1:** Demographic and clinical characteristics of study participants.

	CON (*n* = 14)	Boxers (*n* = 20)	*P*-value
Age (years)	28.1 ± 4.1	29.9 ± 4.9	0.265
Education (years)	15.1 ± 2.3	14.4 ± 2.1	0.380
Professional boxing (years)	NA	6.6 ± 4.4	NA
Participation in boxing (years)	NA	11.0 ± 5.0	NA
Bouts	NA	9.6 ± 8.0	NA
Smoking			
Never smoker, *n* (%)	9 (64.3)	10 (50.0)	0.819
Former smoker, *n* (%)	2 (14.3)	3 (15.0)	
Current smoker, *n* (%)	3 (21.4)	7 (35.0)	
Alcohol			
Never drinker, *n* (%)	0 (0)	4 (20.0)	0.078
Former drinker, *n* (%)	0 (0)	2 (10.0)	
Current drinker, *n* (%)	14 (100)	14 (70.0)	
Physical Activity (MET-minutes)	4,569.9 ± 4,090.5	6,841.6 ± 6,042.7	0.231

*Data are presented as Mean ± SD, or n (%). Abbreviations; CON, healthy controls; NA, not available or not applicable; SD, standard deviation; MET, metabolic equivalent of task*.

Cognitive functions including memory and attention domains were assessed using three cognitive tasks implemented in the Cambridge Neuropsychological Test Automated Battery (CANTAB^®^, Cambridge Cognition Ltd., Cambridge, UK^1^; Sahakian and Owen, 1992). Memory function was measured using the Paired Associate Learning (PAL) task, while attention function was evaluated using the Rapid Visual Information Processing (RVP) task and Intra-Extra Dimensional Set Shift (IED) task. Among 20 professional boxers, two participants refused to participate in cognitive function tests. Therefore, cognitive function tests of 18 boxers and 14 healthy controls were analyzed for this study.

The PAL task measures simple visual memory and visuospatial associative learning. Among the available variables derived from the PAL task, the total number of errors is one of the most frequently used outcomes of the PAL tasks (Smith P. J. et al., 2013; Lenehan et al., 2016). In addition, it has been reported to sensitively measure early cognitive decline in older adults (Junkkila et al., 2012). Lower scores represent better performance. Other outcomes from the PAL task including the number of total trials, the first trial memory scores and the mean errors to succeed were compared between the groups and the results were presented in the Supplementary Table S1. The RVP task assesses the function of sustained attention by measuring the subject’s ability to efficiently detect the target sequence. The outcome measure includes the probability of sensitivity ranging from 0 to 1, which represents signal detection rate. The IED task examines a subject’s ability to attend to specific stimuli as well as flexibility of attention when required. The number of errors was used as an outcome measure.

Raw scores of each of the three tasks mentioned above were converted to standardized *Z* scores using the group mean scores and standard deviation (SD) of the CON group. If necessary, the scores were reversed so that positive *Z* scores to represent better performance. The standardized *Z* score of the adjusted total errors in the PAL task was used to measure memory function and the composite score constructed by averaging *Z* scores of sensitivity in the RVP task and adjusted total errors in the IED task was used to measure attention function.

### Magnetic Resonance Imaging Acquisition

High-resolution structural imaging and magnetic resonance spectroscopy were acquired using a 3.0 Tesla Philips Ingenia MR scanner (Philips Medical System, Bests, Netherlands). For spectroscopic voxel localization and tissue segmentation, high resolution T1-weighted images were acquired using a three-dimensional T1-weighted magnetization-prepared rapid gradient echo sequence with the following parameters: repetition time (TR) = 7.4 ms, echo time (TE) = 3.4 ms, flip angle (FA) = 8°, field of view (FOV) = 220 × 220 mm^2^, slice thickness = 1 mm, number of excitation (NEX) = 1,180 contiguous sagittal slices. GABA+ and Glx levels in the VOI were measured using the Mescher-Garwood point-resolved spectroscopy (MEGA-PRESS) sequence with the following parameters: TR = 2,000 ms, TE = 68 ms; number of signal averages = 320, scan duration = 11 min, water suppression method = multiple optimizations insensitive suppression train, second-order pencil beam shimming. During odd-numbered acquisitions, Gaussian inversion pulse was applied at 1.9 ppm^3^ CH_2_ resonance of GABA, influencing the J-coupled peak at 3.02 ppm (EDIT-ON). The same pulse was provided symmetrically to the other side of the water peak at 7.5 ppm for even-numbered acquisitions (EDIT-OFF).

### Voxel Localization

**Figure 1:**
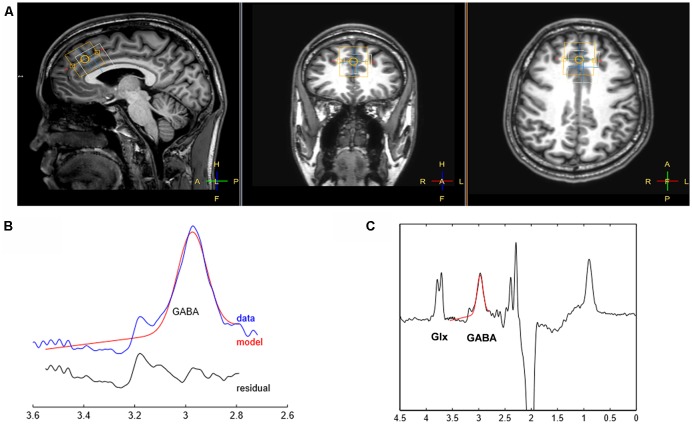
Illustration of voxel placement and sample spectral fitting using Mescher-Garwood point-resolved spectroscopy (MEGA-PRESS). **(A)** The position of voxels in a T1-weighted image. The yellow box represents the voxel of interest (VOI) for γ-aminobutyric acid (GABA) placed on the prefrontal cortex (PFC; VOI: 3 × 3 × 3 cm^3^) while the white box depicts the location where the water signal is acquired. **(B)** The curve-fitting of the GABA peak using Gannet 2.0 toolkit. The red lines in the panels are the best fitted model using a simple Gaussian model of the GannetFit; the blue lines show the GABA-edited spectrum corrected for phase and frequency; the black line represents the residual difference between the curve fit and experimental data, indicated as red and blue lines, respectively. **(C)** The MEGA-PRESS difference spectrum. The Glx and GABA peaks resonate at 3.7 and 3.0 ppm, respectively.

Proton spectra were obtained from the VOI (3 × 3 × 3 cm^3^) located on the PFC (Figure 1). The VOI was positioned along the bicommissural line in the sagittal plane of the T1-weighted image, prescribed anterior to the genu, and along the superior border of the corpus callosum, and centered on the interhemispheric fissure in the axial and coronal planes (Bai et al., 2015). The VOIs were arranged to avoid the lateral ventricles and skull.

### MRS Fitting and Analysis

MEGA-PRESS sequence allows detection of the GABA peak of the spectra by eliminating the signal from creatine. The difference between the “EDIT-ON” and “EDIT-OFF” spectra provides an edited spectrum of GABA (Gao et al., 2013). The detected signal is referred to as GABA+ rather than GABA as the MRS signal at 3.02 ppm acquired using the parameters of MEGA-PRESS is known for containing both macromolecules and homocarnosine (Rothman et al., 1997; Gao et al., 2013). Additionally, we evaluated the co-edited Glx (Glutamate + glutamine) signal at 3.7 ppm in the difference spectra.

The Gannet 2.0 toolkit, a Matlab-based quantitative batch analysis tool for MEGA-PRESS spectra, is used to analyze GABA MEGA-PRESS spectra and quantify GABA+ and Glx (Edden et al., 2014). Gannet contains two modules: GannetLoad and GannetFit. The GannetLoad module is used to process raw time-domain data into a frequency-domain GABA-edited spectrum, apply a line broadening of 3 Hz, and correct for phase and frequency by removing artifacts due to motion and scanner drift. Through nonlinear least-squares fitting of the spectra, GannetFit applies a single-Gaussian model to estimate the area under the edited GABA signal at 3 ppm as well as the creatine (Cr) signal at 3 ppm. Quantitative results are then expressed as the ratios of GABA+ and Glx relative to Cr (Ng et al., 2014). The GABA+/Cr ratio and the Glx/Cr ratio were used to represent the GABA+ and Glx level, respectively. The overall FitError reflecting the signal-to-noise ratio is estimated by dividing the SD of the fitting residual with the amplitude of the fitted peaks, using the GannetFit module. Only spectra with a relative FitError of GABA+ or Glx below 10% were used for the statistical analysis. The mean FitError of GABA+ and Glx was estimated for two groups (Boxers; GABA+, 7.07 ± 1.43%: Glx, 5.51 ± 0.49%; CON; GABA+, 7.14 ± 0.82%: Glx, 5.52 ± 0.38%). There were no significant differences in FitError in GABA+ and Glx between the two groups (*P* = 0.865 for GABA+, *P* = 0.827 for Glx, respectively). Also, the fraction of gray matter within the VOI did not significantly differ between the two groups (52.5 ± 0.02% vs. 51.5 ± 0.03%, *P* = 0.240).

### Statistical Analysis

For the sample-size calculation, the effect size of group-difference in the GABA+/Cr ratio was estimated based on approximately a 10% differences since there were no prior studies showing a significant group-differences of GABA levels in rmTBI patients. Based on an alpha level of 0.05, a minimum of 14 individuals per group was required. Since we expected that higher drop-out rate in the Boxers compared to the CON group (30% of drop-out for the Boxers Vs. 10% for the CON group), we initially planned to enroll 16 individuals for the CON and 19 individuals for the Boxers group.

Demographic characteristics were compared between the two groups using independent *t*-tests and chi-square tests for continuous and dichotomous variables, respectively. Group differences in memory and attention functions, as well as the GABA+/Cr and Glx/Cr, were also assessed using general linear model (GLM) with age as a covariate. Eta squared (*η*^2^) was used to estimate the effect size. Spearman correlation analysis was performed to evaluate levels of GABA+ and Glx in relation to cognitive functions for both Boxers and CON. Statistical significance was assessed by a permutation-based test with a threshold of *P* < 0.05. A permutation-adjusted *P* value was computed based on the proportion of permutations with *P* values under the null distribution that was greater than the observed values from the actual data set (Westfall et al., 1993). Correction for multiple comparisons was performed using a bootstrap-based test (Westfall and Young, 1993). Data were tested by running 10,000 synthesized resampling with a threshold of *P* < 0.05. All statistical analyses were performed using STATA software package, version 13.0 (StataCorp, College Station, TX, USA).

## Results

### Demographic and Clinical Characteristics

The Boxers and CON groups showed no significant differences in terms of demographic characteristics which include age and educational level. The amount of physical activity did not significantly differ between the Boxers and the CON groups (Table 1).

### Group Differences in Neurometabolite Measures

**Figure 2:**
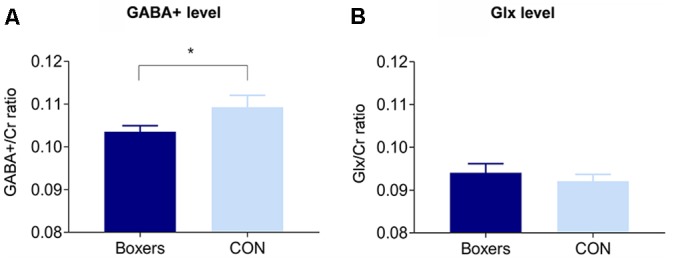
Between-group differences in the GABA+/Cr and Glx/Cr in the PFC. **(A)** The GABA+/Cr was significantly lower in the Boxers group compared to the CON group (*z* = −2.21, *permutation-adjusted P* = 0.032). **(B)** There was no significant difference in the Glx/Cr between the Boxers and CON groups (*z* = 0.88, *permutation-adjusted P* = 0.386). Error bars indicate standard errors. *Indicates *P* < 0.05.

The GABA+/Cr was significantly lower in the Boxers relative to the CON (*z* = −2.21, *permutation-adjusted P* = 0.032; Figure 2A), which remained significant after removing the two subjects in the Boxers group who refused to take part in the cognitive tests (*z* = −2.31, *permutation-adjusted P* = 0.023). However, there was no significant difference in the Glx/Cr between the two groups (*z* = 0.88, *permutation-adjusted P* = 0.386; Figure 2B).

### Group Differences in Cognitive Measures

**Figure 3:**
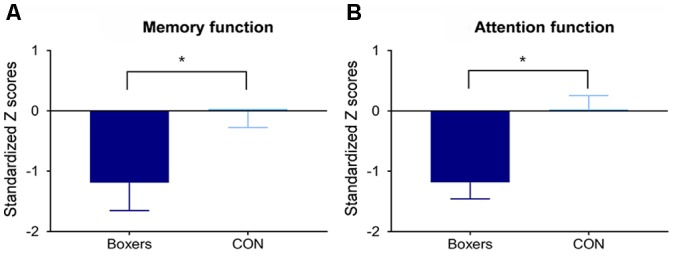
Between-group difference in the memory and attention functions. Standardized *Z* scores of memory and attention function were calculated using the corresponding means and standard deviations (SDs) of the control group. Error bars indicate standard errors. The Boxers group showed poorer performance **(A)** in the memory domain (*z* = −2.01, *permutation-adjusted P* = 0.045) and **(B)** in the attention domain (*z* = −2.92, *permutation-adjusted P* = 0.006) compared to the CON group. *Indicates *P* < 0.05.

The Boxers group showed lower performance in the memory domain (*z* = −2.01, *permutation-adjusted P* = 0.045; Figure 3A) and attention domain (*z* = −2.92, *permutation-adjusted P* = 0.006; Figure 3B) as compared to the CON group.

### Relationships Between Cognitive and Neurometabolite Measures in the Boxers Group

**Figure 4:**
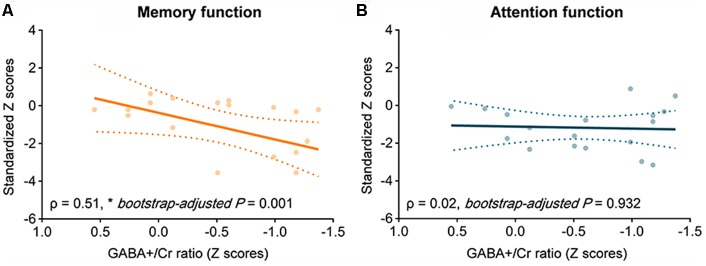
The relationship between GABA+/Cr and cognitive performance in the Boxers group. Scatter plots with the line of best fit showing correlations between GABA+/Cr and standardized *z* scores of memory performance measured using the Paired Associate Learning (PAL) task. Spearman correlation analysis was used to measure correlations at the significance level of 0.05. In the Boxers group, there was a positive correlation between GABA+/Cr and performance **(A)** in memory task (*ρ* = 0.51, *z* = 3.21, *bootstrap-adjusted P* = 0.001) but **(B)** not in attention task (*ρ* = 0.02, *z* = 0.09, *bootstrap-adjusted P* = 0.932).

The GABA+/Cr in the prefrontal VOI was positively associated with memory performance (*ρ* = 0.51, *z* = 3.21, *bootstrap-adjusted P* = 0.001; Figure 4A) but not with attention (*ρ* = 0.02, *z* = 0.09, *bootstrap-adjusted P* = 0.932; Figure 4B) in the Boxers group. On the other hand, the Glx/Cr did not show any significant correlation with memory performance (*ρ* = 0.25, *z* = 0.64, *bootstrap-adjusted* *P* = 0.522) or attention function (*ρ* = −0.05, *z* = −0.20, *bootstrap-adjusted* *P* = 0.841) in the Boxers group.

### Relationships Between the Severity or Frequency of rmTBI and Neurometabolite Measures in the Boxers Group

There were no significant correlations between the GABA+/Cr and the number of bouts (*ρ* = −0.13, *z* = −0.57, *bootstrap-adjusted P* = 0.571) as well as the GABA+/Cr and the duration of boxing (*ρ* = 0.03, *z* = 0.10, *bootstrap-adjusted P* = 0.918) in the Boxers group. There were also no significant correlation between the Glx/Cr and the number of bouts (*ρ* = 0.11, *z* = 0.44, *bootstrap-adjusted P* = 0.659) as well as the GABA+/Cr and the duration of boxing (*ρ* = 0.09, *z* = 0.33, *bootstrap-adjusted P* = 0.741) either.

## Discussion

To the best of our knowledge, the current study provides the first evidence of the role of GABA levels in the PFC and its association with memory dysfunction in professional boxers with rmTBI. The Boxers group, relative to the CON group, demonstrated lower GABA+/Cr in the PFC as well as poor performances in memory and attention functions. Lower GABA+/Cr was significantly associated with memory dysfunction in the Boxers group. Given that GABA plays a crucial role in human behavioral functions as the major inhibitory neurotransmitter in the adult brain, the current findings may imply that lower GABA levels found in professional boxers may contribute to the development of memory dysfunction following rmTBI.

In alignment with the current key findings regarding low GABA levels in boxers with rmTBI, previous animal studies have suggested that the imbalance between GABA and Glx levels may be one of the pathophysiological mechanisms underlying the chronic cognitive and behavioral changes following to rmTBI (Guerriero et al., 2015). The lower GABA levels found in the Boxers group may reflect a loss of GABA-producing (GABAergic) interneurons, a decrease in GABA synthesis, or alterations of cycling of GABA and Glx in astrocytes (Rae, 2014). GABAergic interneurons are categorized into the subtypes based on the molecular markers including the calcium-binding peptide parvalbumin (PV)-expressing interneurons and the somatostatin-expressing interneurons (Fino et al., 2013). Previous studies on mTBI have suggested that reduced GABA levels related to mTBI in the chronic phase may have originated from the preferential downregulation of PV-positive GABA interneurons (Huusko and Pitkänen, 2014; Guerriero et al., 2015; Vascak et al., 2018). Therefore, the resultant disruption of inhibitory neurotransmission by the downregulation of PV-positive GABAergic interneurons (Sohal et al., 2009) may increase excitotoxic neuronal injury, particularly in the brain regions involved in learning and executive functioning such as the prefrontal lobes and hippocampus (Stuss, 2011; Giza and Hovda, 2014). Such neurometabolic disturbances related to rmTBI have also been reported to play an important role in developing various types of chronic cognitive and behavioral symptoms (Gibson et al., 2010; Guerriero et al., 2015).

It is noteworthy that there were no statistical differences of the Glx/Cr ratio between the Boxers and CON groups. This result is partially consistent with previous findings which indicate that glutamate levels initially decrease during the acute stage and gradually return to the normal range in the chronic phase, at the time of  6 months after the exposure to rmTBI (Henry et al., 2010). Given that the duration of exposure to boxing-related rmTBI was more than 2 years for the participants recruited in the current study, it is plausible that alterations in Glx levels might have been present in the Boxer group prior to the study, and then normalized at the time of assessment.

Consistent with previous studies reporting that attention and memory dysfunctions are core clinical features of rmTBI-related CTE (Montenigro et al., 2015; Wilde et al., 2016), the current findings also demonstrated that the Boxers showed impaired performance in memory and attention tasks, as compared to the CON group. Indeed, the cognitive dysfunctions found in Boxers of the current study were not severe enough to affect their daily functioning or quality of life. However, studies have shown that such subtle decline may eventually increase the risk for more pronounced cognitive deficits later in life (De Beaumont et al., 2009, 2012). Therefore, our results may suggest that rmTBI, even in young athletes who are actively participating in boxing, could potentially impact cognitive dysfunctions as a cumulative effect.

Interestingly, there was a notable positive relationship between memory performance and the GABA+/Cr of the PFC in the Boxers group, which can be assumed that altered GABA levels in the Boxers group may mediate memory dysfunctions following rmTBI. Consistent with this finding, previous studies have reported that reduced GABA levels in the PFC were associated with cognitive dysfunctions in age-related cognitive decline (Gao et al., 2013) as well as in neuropsychiatric diseases such as depression, schizophrenia and Alzheimer’s disease (Northoff and Sibille, 2014; Porges et al., 2017). Since PV-positive GABAergic neurons in the PFC are known to be associated with the generation of gamma oscillations during memory consolidation (Xia et al., 2017) as well as working memory and cognitive flexibility (Murray et al., 2015), a reduction in PV-positive GABAergic interneurons in the PFC of individuals with rmTBI may play a role in memory dysfunctions. However, we did not find a significant association between the GABA+/Cr in the PFC and performance on the attention task in the Boxers group. The human visual attentional function requires both top-down as well as bottom-up attention processes (Buschman and Miller, 2007; Li et al., 2010). Thus, the lack of association between attention and the GABA+/Cr could be partially explained by the fact that RVP and IED task in our study are associated with not only top-down attentional function which is mainly involved in the PFC but also bottom-up attention, mainly controlled by posterior parietal cortex. In order to identify a clear link between TBI-related GABA disturbance and attention deficits, future studies would be necessary to be performed in both the PFC and posterior parietal cortex.

As a final point, it should be noted that there was no significant correlation between the GABA+/Cr and the number of bouts as well as the GABA+/Cr and the duration of boxing. This may suggest that the GABA+/Cr ratio may not a state marker that reflects the severity or frequency of rmTBI but a trait marker that may represent the cognitive dysfunctions related to rmTBI in professional boxers. Consistent with our findings, reduced GABA level in the brain has been regarded as a trait marker for many neuropsychiatric disorders such as depression or bipolar disorders (Petty, 1995; Bhagwagar et al., 2007). However, given that deficits in GABA-mediated cortical inhibition could be both trait and state markers depending on the type of disrupted GABAergic system such as GABA_A_ or GABA_B_ (Ruiz-Veguilla et al., 2017), replication studies of longitudinal design with consideration of different type of GABAergic system could be helpful to determine whether the GABA+/Cr ratio could be a state and/or a trait marker in the professional boxers.

Potential limitations in interpreting the current findings should be noted. First, we included only 14 individuals for the CON, although we originally planned to recruit 16 for the CON. Although the group-difference in the GABA+/Cr was also significant after permutation-based test (*z* = −2.21, *permutation-adjusted P* = 0.032) and the effect size of the group-difference in the GABA+/Cr was medium to large (*η*^2^ = 0.14; Cohen, 1973; Levine and Hullett, 2002), further studies with a larger sample size are warranted in order to obtain a more robust finding. Second, the observed alterations of GABA in the Boxers may have been driven by differences in macromolecules or homocarnosine since the detected GABA signal contains both macromolecules and homocarnosine. Although the differences were interpreted as alterations in GABA concentration in this study, further studies that examine the isolated effects of GABA are required. Third, a causal relationship between rmTBI and GABA levels could not be evaluated as the design of this study was cross-sectional. Also, the current study measured the GABA level of each participant once, and as such does not account for the trajectory of metabolic changes in relation to rmTBI. Finally, although the age distribution was included in the models as a covariate, the particular effects of rmTBI during adolescence have not been considered in this study. Since the participants in the Boxers group varied in ages (20–37 years) and they had been professionally boxing for at least 2 years, some of young professional boxers may have suffered rmTBI during adolescence. While experience of rmTBI in adolescence may increase vulnerability to the neurobiological sequelae related rmTBI (Moser and Schatz, 2002), on the other hand, neuroplasticity in early life may exert potential protective effects on the long-term outcomes associated with rmTBI in adolescence (Sariaslan et al., 2016). Therefore, further studies would be necessary to consider the particular contribution of rmTBI during adolescence to neurobiological and cognitive changes in the course of professional boxing.

The current study findings of positive associations between lower GABA levels and memory function may provide further insight that lower GABA levels could be regarded as a neurochemical mechanism underlying cognitive impairment in professional boxers with rmTBI. The findings from this study further support the development of intervention and prevention strategies of rmTBI, by suggesting that strategies to increase prefrontal GABA levels during pre-clinical stages may improve the neurocognitive outcomes of those with rmTBI as in professional boxers.

## Ethics Statement

This study was carried out in accordance with the recommendations of Institutional Review Board of Ewha Womans University with written informed consent from all subjects. All subjects gave written informed consent in accordance with the Declaration of Helsinki. The protocol was approved by the Institutional Review Board of Ewha Womans University.

## Author Contributions

GK contributed to the conception, design of the study, organized the data base, performed the statistical analysis and wrote the first draft of the manuscript. IL, HJ, SP, HH, JK and JYK organized the database. RE organized the database and interpreted the results. IL and SY contributed to the conception, design of the study, organized the data base, interpreted the results and finally approved the submitted manuscript. All authors read and approved of the submitted version of the manuscript.

## Conflict of Interest Statement

The authors declare that the research was conducted in the absence of any commercial or financial relationships that could be construed as a potential conflict of interest.

## Footnotes

^1^www.cantab.com
