# Supplementary material for: Low Prefrontal GABA Levels Are Associated With Poor Cognitive Functions in Professional Boxers
Source: Front Hum Neurosci. 2019 Jun 11;13:193. doi: 10.3389/fnhum.2019.00193 (PMC6579878; doi:10.3389/fnhum.2019.00193)
Supplement: Supplementary file 1 [file Table_1.docx]

**Supplementary Table 1.** Between group differences of other outcomes of PAL task

|  | **CON (n=14)** | **Boxers (n=18)** | ***P-value*** |
| --- | --- | --- | --- |
| ***Memory*** |  |  |  |
| **Paired Associate Learning (PAL)** |  |  |  |
| **Memory scores-first trial** | 15.4 ± 2.9 | 12.6 ± 3.3 | 0.015 |
| **Total trials ^a^** | 9.1 ± 3.0 | 11.2 ± 3.6 | 0.088 |
| **Mean errors to succeed ^a^** | 2.3 ± 1.7 | 4.8 ± 4.3 | 0.043 |

^a^ Lower scores represent better performance

Abbreviations: CON, healthy controls
